# Hormone Receptor‐Dependent Correlations Between Angiopoietins and VEGF‐C in Primary Breast Cancer: Insights Into Lymphangiogenic Biomarkers

**DOI:** 10.1002/cnr2.70101

**Published:** 2025-05-09

**Authors:** Vahid Montazeri, Parisa Varshosaz, Ashraf Fakhrjou, Saeed Pirouzpanah

**Affiliations:** ^1^ Drug Applied Research Center Tabriz University of Medical Sciences Tabriz Iran; ^2^ Molecular Medicine Research Center Tabriz University of Medical Sciences Tabriz Iran; ^3^ Department of Thoracic Surgery, Faculty of Medicine Tabriz University of Medical Sciences Tabriz Iran; ^4^ Surgery Ward Nour‐Nejat Hospital Tabriz Iran; ^5^ Department of Pathology School of MedicineTabriz University of Medical Sciences Tabriz Iran

**Keywords:** angiopoietin, biomarker, breast cancer, diagnostic accuracy, hormone receptor, lymph node metastasis, lymphangiogenesis, vascular invasion, VEGF‐C

## Abstract

**Background:**

Biomarkers of angiogenesis and lymphangiogenesis have been explored in cancer prognostic models; however, their potential role in assessing local tumor invasiveness remains poorly understood.

**Aims:**

This study aimed to evaluate the correlations of angiogenic biomarkers, specifically the angiopoietin (ANG)‐Tie system and vascular endothelial growth factor‐C (VEGF‐C), with lymphangiogenesis and the related histopathological characteristics in Iranian women with breast cancer.

**Methods and Results:**

In this consecutive case series (*n* = 149) from the Breast Cancer Risk and Lifestyle (BCRL) study, plasma levels of pro‐angiogenic factors, including VEGF‐C, ANGs, and Tie‐2, were assessed using ELISA. Clinicopathological data were collected, excluding stage IV cases to focus on patients with localized disease. Axillary lymph node metastasis (ANLM), and vascular invasion (VI) were common in the study population, occurring in 61.5% and 77.6% of cases, respectively (*p* < 0.01). Estrogen receptor‐positive (ER^+^) tumors were observed in 89.1% of ANLM^+^ participants, while human epidermal growth factor receptor‐2‐positive (HER‐2^+^) tumors were identified in 22.8% of patients with ALNM. Plasma levels of ANG‐1 (*r* = 0.19) and VEGF‐C (*r* = 0.29) were positively correlated with the ALNM ratio (*p* < 0.05). Multivariate analysis in patients with grade II tumors revealed significant inverse correlations between VEGF‐C and angiogenic biomarkers, including ANG‐2 (*β* = −0.25), the ANG‐2/Tie‐2 ratio (*β* = −0.28), and the (ANG‐1 + ANG‐2)/Tie‐2 ratio (*β* = −0.29) (*p* < 0.05). Receiver operating characteristic (ROC) curve analysis indicated that ANG‐2 could effectively assess ALNM status, with an optimal cutoff of 3.39 pg/mL, identifying ALNM in 66.0% of patients with low VEGF‐C levels (95% CI: 0.54–0.78), increasing to 68.0% when combined with ANG‐1 as the ANGs/Tie‐2 ratio (95% CI: 0.56–0.80). In ER^+^ tumors, high plasma ANG‐2 levels were observed (*p* < 0.05). Significantly higher levels of the (ANG‐1 + ANG‐2)/VEGF‐C ratio were noted in patients with VI+ (*p* < 0.05). Findings descriptively highlighted ER^+^ status as a common characteristic in VI^+^ and ALNM^+^ tumors. In HER‐2^+^ patients, both ANG‐1 and the (ANG‐1 + ANG‐2)/Tie‐2 ratio showed inverse correlations with VEGF‐C, while in ER^−^ breast cancer patients, ANG‐2 was inversely correlated with VEGF‐C.

**Conclusion:**

These findings provide new insights into the inverse correlation between plasma levels of ANGs and VEGF‐C, particularly in cases with positive ALNM, underscoring the role of hormone receptor‐dependent characteristics. The integration of the triple angiogenic biomarkers ANG‐2/Tie‐2/VEGF‐C within the tumor microenvironment, combined with the regulatory influence of hormonal receptors, merits further investigation as a potential biomarker panel for identifying lymphatic anomalies and VI positivity in breast cancer patients.

## 1 Introduction

Lymphangiogenesis, the formation of new lymphatic vessels, is a crucial step in malignant pathological transformations and tumor metastasis [1]. This process shares similarities with angiogenesis and is regulated by a complex balance of pro‐ and anti‐angiogenic growth factors [2]. These factors influence tumor cell growth and progression, often accelerating the transition to advanced metastatic stages [3]. Cancer cells secrete pro‐angiogenic growth factors that drive endothelial cell proliferation, vessel sprouting, migration, and enlargement, ultimately contributing to the development of new vessels [4]. Among the diverse angiogenic factors, vascular endothelial growth factor (VEGF) and angiopoietins (ANGs) are central to angiogenesis, though their roles in inducing lymphangiogenesis differ significantly [5, 6]. The VEGF‐C, in particular, plays a pivotal role in the formation and remodeling of lymphovascular structures, with its effects mediated through specific VEGF receptors [2]. VEGF‐C binding to VEGFR‐2 is associated with both tumoral angiogenesis and lymphangiogenesis, whereas VEGF‐C binding to VEGFR‐3 specifically drives lymphangiogenesis [2, 7]. Epidemiological studies have demonstrated that VEGF‐C protein levels correlate with lymph node invasiveness in cancer [8, 9], highlighting its potential as a diagnostic biomarker for lymphatic progression in breast tumors.

Angiopoietins, such as ANG‐1 and ANG‐2, play a crucial role in regulating angiogenesis by interacting with the Tie‐1 (tyrosine kinases receptor with immunoglobulin‐like and EGF‐like domain 1) and Tie‐2 receptors [10]. ANG‐1, an agonist ligand for Tie‐2, supports endothelial cell integrity, migration, and blood vessel maturation [11]. In contrast, ANG‐2, which competes with ANG‐1 for Tie‐2 binding, triggers Tie‐2 autophosphorylation [12], leading to the destabilization of endothelial cell junctions and promoting vessel sprouting and branching [10, 11]. In addition, the dynamic balance between ANG‐1 and ANG‐2 (e.g., ANG‐1/ANG‐2) during carcinogenesis highlights the critical role of VEGF‐C in endothelial cell proliferation [13]. When VEGF levels are low, ANG‐2 can induce the regression of vascular formation and exhibit antagonistic effects [11]. The intricate regulation of vessel formation by ANGs dependent on VEGF levels, with the ANG‐1/ANG‐2 ratio playing a crucial in determining whether endothelial cells proliferate to form new blood vessels or regress existing ones [14].

While various growth factors are known to promote lymphangiogenesis [13], the interactions between ANGs and VEGF‐C in relation to clinical factors—such as tumor size [15], grade [16], and migration‐related characteristics like vascular or lymphatic invasion—have been less extensively studied [17]. Some studies have linked elevated VEGF levels with advanced breast cancer features, including larger tumor sizes and axillary lymph node metastasis (ALNM) [18, 19]. While some studies have indicated that VEGF‐C and ANGs function together in promoting lymphangiogenesis [19, 20], a comprehensive understanding of the ANGs/Tie/VEGF‐C interaction system in pathologic lymphangiogenesis still requires further investigation [21].

The estrogen receptor (ER) and progesterone receptor (PR) are well‐established tumor markers clinically used to predict the likelihood of response to endocrine therapy [22, 23, 24, 25]. Intracellular ER‐dependent signaling is a potential growth‐promoting factor in ER^+^ tumors [23], contributing to angiogenesis by enhancing endothelial cell proliferation [24, 25]. Evidence indicates that ER signaling response elements are located in the promoters of *VEGF* and *ANG* genes [26, 27]. However, no epidemiological study has yet explored the role of hormonal receptor (HR; i.e., ER and PR) expression in the inter‐relationship of angiogenic factors in breast cancer.

The upregulation of the epidermal growth factor receptor (EGFR‐2 or HER‐2) is a well‐recognized histopathologic marker of breast cancer, often associated with poor prognosis [8]. In vitro investigations using breast cancer cell lines have demonstrated that VEGF‐C can induce HER‐2‐dependent tumor cell migration [28]. Recent studies also indicate a correlation between elevated HER‐2 expression and increased VEGF‐C levels, reinforcing the hypothesis that HER‐2‐mediated angiogenic interactions might be linked to lymphangiogenesis [8, 29].

Investigating angiogenic biomarkers as potential indicators for identifying patients at risk of metastasis offers a promising research direction. Additionally, the influence of specific hormone‐receptor profiles on variations in angiogenic biomarkers remains largely unexplored. This study therefore aims to investigate the associations among plasma levels of angiopoietins, Tie‐2, and VEGF‐C, and their potential links to lymphangiogenesis and key histopathological features in Iranian women with primary breast cancer.

## 2 Materials and Methods

### 2.1 Study Population

The present study population is drawn from the Breast Cancer Risk and Lifestyle (BCRL) study, a prospective, consecutive case series initiated in 2009 that targets females diagnosed with primary breast cancer in Northwestern Iran. Participants were recruited from surgical wards of Nour‐Nejat, Shams, and Imam‐Reza referral hospitals, along with the oncology departments of Shahid‐Ghazi Hospital in Tabriz, Iran. This multicenter study was designed to explore potential lifestyle‐related risk factors for breast cancer within the population of Northwestern Iran. The current recruitment comprises 149 newly diagnosed women with histologically confirmed primary breast cancer admitted to the surgical ward of the Nour‐Nejat Hospital, Tabriz, Iran. Enrolment and data collection took place pre‐surgery (mastectomy or partial mastectomy) between October 2014 and April 2016. The mean age at diagnosis was 46.4 ± 8.6 years (range: 30–75 years; median age: 46.0 years). The inclusion criteria for this study comprised women diagnosed with non‐metastatic breast cancer (clinical stage I–IIIA), who provided written informed consent and had no history of other cancers or benign breast diseases. The exclusion criteria included pregnancy, breastfeeding, and chronic use of medications that could affect cancer metabolism and proliferation. Other medical conditions leading to exclusion were previous oncologic surgeries, and prior adjuvant or neoadjuvant therapies (e.g., chemotherapy, radiotherapy, and hormonal therapy), which were assessed to ensure the selection of primary malignancy. Additionally, participants with chronic inflammatory diseases (e.g., cardiovascular disease, and type II diabetes), or those using anticonvulsants, contraceptives, or hormone replacement therapy within the 2 years prior to the study were also excluded. Further details on the exclusion criteria can be found in previous publications [23, 30, 31, 32, 33, 34, 35].

### 2.2 Ethics Statement

Prior to enrollment, participants received a verbal description of the study and its ethical considerations, accompanied by a written consent form. The study design and procedures were compiled with the Ethical Guidelines for Observational Studies [32, 36]. The research protocol which outlined the methodology, study subjects, sample size, data collection, biochemical tests, analysis, and ethical considerations, was reviewed and approved by the Ethical Committee of Tabriz University of Medical Sciences (ethical code: 5‐4‐8327). This report adheres to the STROBE guidelines for observational studies [37].

### 2.3 Interview

Data on sociodemographic factors, reproductive history, and lifestyle characteristics, were collected through face‐to‐face interviews in hospital settings. This include age, occupation, education levels, reproductive factors (e.g., lactation history), medical history, family history of breast cancer, physical activity, and smoking habits. Anthropometric measurements, including waist circumference (cm), height (cm), and weight (kg), were taken, and body mass index (BMI, kg/m^2^) was calculated subsequently.

### 2.4 Laboratory and Biochemical Analysis

Venous blood samples were collected from enrolled participants following overnight fasting and prior to surgery, using EDTA tubes (Becton Dickinson Vacutainer Systems, Plymouth, UK). The samples were promptly centrifuged at 3000 × g for 10 min (Sanyo‐Gallenkamp Ltd. Loughborough, UK), and the plasma supernatant was aliquoted into sterilized tubes, and stored at −80°C until laboratory analyzes.

Commercial enzyme‐linked immunosorbent assay (ELISA) reagents (Cusabio, China: Cat. no. CSB‐EL001706HU for ANG‐1, Cat. no. CSB.E04500h for ANG‐2, and Cat. no. CSB‐EL023375HU for Tie‐2; eBioscience, United Kingdom: Cat. no. BMS297/2 for VEGF‐C) were used to measure plasma levels according to the respective kit instructions. Intraobserver and interobserver coefficient variations (CVs) for ANGs (ANG‐1, and ANG‐2), and Tie‐2 were < 8% and < 10%, respectively. Serum VEGF‐C concentration measurement exhibited a CV of 6.7% within observers and 12% between observers. Each biomarker measurement for every individual was conducted in triplicate simultaneously within a single laboratory run.

### 2.5 Pathological and Immunohistochemical (IHC) Data

Specific antibodies were employed for IHC staining of microtome sections from paraffin‐embedded tissue specimens, conducted overnight at 8°C in the Azerbaijan Sharghi Pathology Laboratory, Tabriz, Iran. Cell staining percentages were evaluated using a binocular microscope (Zeiss KF2 binocular, Germany). HER‐2 (HER‐2/neu) was considered positive if the membrane or membrane plus cytoplasmic staining by the antibody (A0485, 1/200; Dako Denmark A/S, Glostrup, Denmark) exhibited weak staining < 10% of tumor cells or higher intensity in ≥ 10% of tumor cells [38]. The IHC analyzes for ER and PR were conducted using Clone ID5 (Dako Denmark A/S, Glostrup, Denmark) and Clone PgR636 (Dako Denmark A/S, Glostrup, Denmark), respectively. A negative status for ER and PR protein expression (i.e., HR) was defined as staining of less than 5% [36].

Pathologic records were carefully reviewed to collect data on histopathological subtypes, tumor size, tumor grade, vascular and lymphatic invasions, ALNM, and IHC results for each patient. Breast tumor with lymphatic invasion (LI+) represents a significant risk factor for the progression of tumor spread to the lymph nodes [39], and is thus anticipated to be closely associated with ALNM status.

The ALNM ratio, calculated by dividing the involved ALNMs by the total number of surgically dissected axillary lymph nodes, is proposed as a new quantitative index for interpreting tumor infiltration within lymphatic vessels in breast cancer.

### 2.6 Statistical Analysis

All statistical analyzes were conducted using the SPSS software (version 23.0; SPSS Inc. Chicago, IL, USA). The chi‐square test was employed to evaluate categorical variables, while independent sample *t*‐test was used to compare continuous variables. Log transformations were applied to all continuous variables except Tie‐2. Each plasma biomarker was categorized based on the median value determined from the study population. To analyze the association between HR status and biomarker levels, odds ratios (OR) and 95% confidence intervals (95% CI) were calculated. One‐way analysis of variance (ANOVA) was utilized to compare variances among multi‐stratum variables. Adjusted models included age at diagnosis (year), BMI (kg/m^2^), number of live births (*n*), and pathological disease grade (І, ІІ, and ІІІ), as main confounding factors. The standardized correlation coefficient value (*β*) was calculated separately for the association of the studied biomarkers with plasma VEGF‐C concentrations across HR status, and the categories of pathological features, including tumor grade, ALNM, VI, and LI. Receiver operating characteristic (ROC) curves were generated to assess ALNM involvement in breast cancer patients based on lymphangiogenic biomarkers under study. For predicting the binary classification of ALNM status, the ROC curve plotted test sensitivity on the *y*‐axis against the false‐positive error rate (1‐specificity) on the *x*‐axis [34, 40]. The ROC curve accounts for the positive likelihood ratio, calculated as sensitivity divided by the false‐positive error rate [40]. Additionally, Youden's index was used to identify the optimal cutoff point for lymphangiogenic biomarkers in detecting ALNM involvement in breast cancer patients. The accuracy of the test biomarker was evaluated using the area under ROC curve (AUC) [34, 35, 40], which represents the overall ability of the test biomarker to distinguish true positive from true negative regarding ALNM. An AUC value between 0.5 and 1.0 is considered suitable [34, 35, 40]. A *p value* less than 0.05 (two‐sided) was regarded as statistically significant.

## 3 Results

### 3.1 General Characteristics

The demographic and histopathologic characteristics of 149 primary breast cancer patients are summarized in Table 1. The mean age at diagnosis was 46.4 ± 8.6 years. Among the study participants, 66.0% (95 out of 144) were premenopausal, while 34.0% (49 out of 144) were postmenopausal. Pathological grade ІІ tumors comprised 80.6% of the cases (112 out of 139; *p* < 0.01), and invasive ductal carcinoma (IDC) was the predominant histopathologic subtype, accounting for 95.0% of cases (114 out of 120, with 29 records missing data; *p* < 0.01). The proportion of ER^+^ tumors was 91.3%, and PR^+^ tumors constituted 88.5%. The HER‐2^+^ tumors comprised 19.4% of the study population (*p* < 0.01).

**TABLE 1:** Histopathologic and general characteristics of participants with breast cancer (*n* = 149).

Variables	Number of patients (n)	Relative frequency (%)	*p* ^a^
Age at diagnosis			
Mean ± S.D.	147	46.4 ± 8.6	
< 46	75	51.1	
≥ 46	72	48.9	0.56
Histopathology			
Ductal	114	95.0	
Others	6	5.0	< 0.01
Histopathological grade			
І	16	11.5	
ІІ	112	80.5	
ІІІ	11	8.0	< 0.01
Tumor size			
< 2.5	42	32.3	
2.5–4	54	41.6	
≥ 4	34	26.1	0.09
Receptor status			
ER			
Positive	136	91.3	
Negative	13	8.7	< 0.01
PR			
Positive	131	88.5	
Negative	17	11.5	< 0.01
HER‐2			
Positive	29	19.5	
Negative	120	80.5	< 0.01
Vascular invasion			
Positive	90	77.6	
Negative	26	22.4	< 0.01
Lymphatic invasion			
Positive	63	60.6	
Negative	41	39.4	0.03
ALMN			
Positive	88	61.5	
Negative	55	38.5	< 0.01
Menopausal status			
Premenopause	95	65.9	
Postmenopause	49	34.1	< 0.01
Number of live birth			
< 2	25	17.5	
≥ 2	118	82.5	< 0.01
Number of lactation			
< 2	29	20.7	
≥ 2	111	79.3	< 0.01
BMI (kg/m^2^)			
< 20	4	3.0	
20–24.9	18	13.7	
≥ 25	110	83.3	< 0.01

*Note:* Some missing data existed in histopathologic variables.

Abbreviations: ALNM, axillary lymph node metastases; BMI, body mass index; ER, estrogen receptor; HER‐2, human epidermal growth factor receptor 2; PR, progesterone receptor.

^a^ The *p value* was obtained using the chi‐square test.

Mean plasma levels of angiogenic factors in breast cancer patients (*n* = 149) were as follows: ANG‐1: 4.9 ± 7.4 ng/mL (reference range: 0.6–6.0 ng/mL) [41], ANG‐2: 4.0 ± 5.5 pg/mL (reference range: 0.5–3.0 pg/mL) [41], Tie‐2: 1.7 ± 0.8 ng/mL (reference range:10.0–92.0 ng/mL) [41], and VEGF‐C: 0.1 ± 0.2 ng/mL (reference range: 0.04–7.75 ng/mL) [41]. Notably, ANG‐2 concentrations exceeded the reference range (*p* < 0.01) [41].

### 3.2 Lymphovascular Involvement and Lymphangiogenic Biomarkers

**FIGURE 1:**
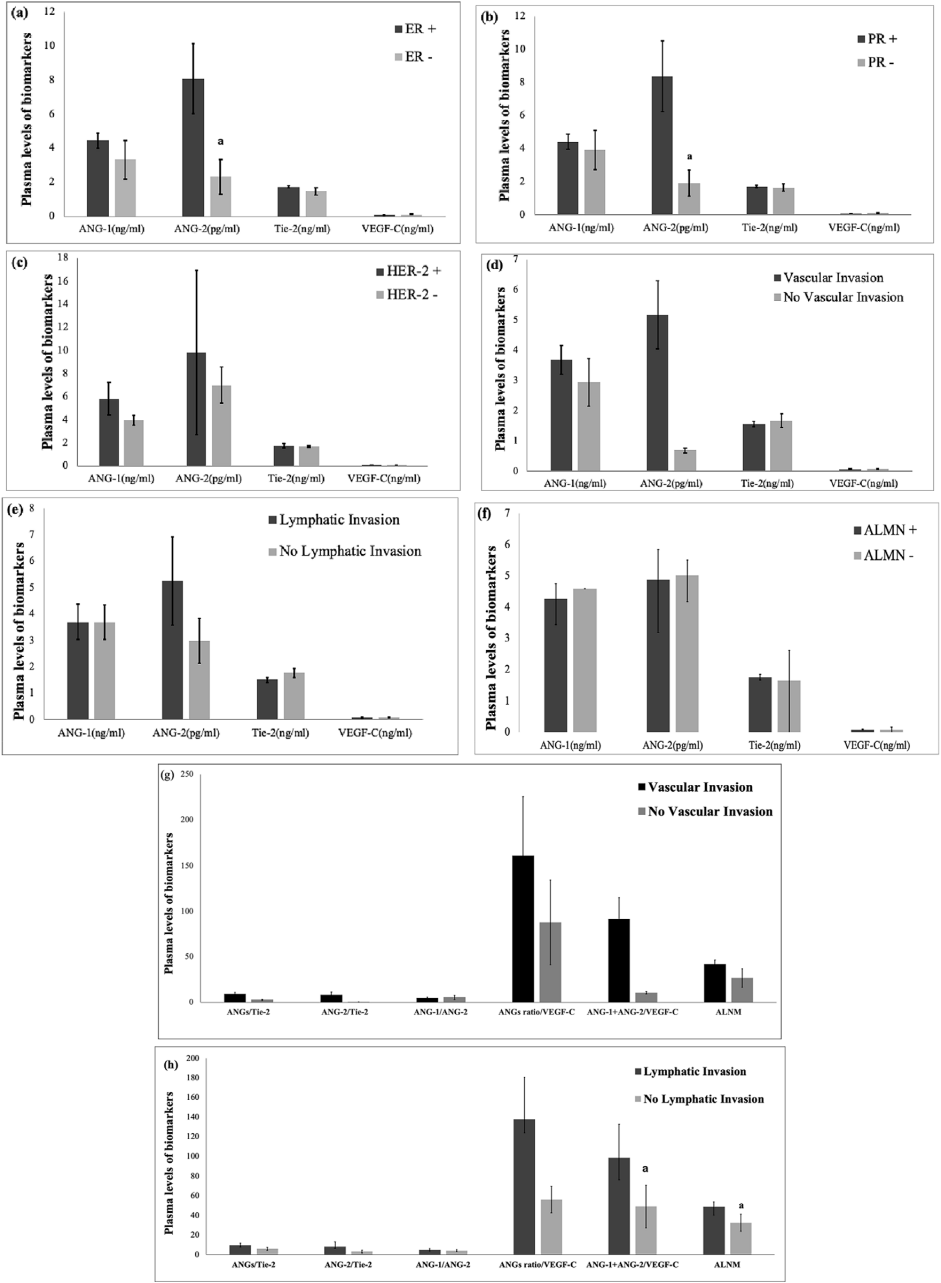
Comparison of angiogenic plasma levels across pathological characteristics. This figure compares plasma biomarker levels based on the presence and absence of hormonal receptors (a–c; *n* = 149), vascular invasion (d; *n* = 116), lymphatic invasion (e; *n* = 104), and axillary lymph node metastasis [ALNM; f (*n* = 143)]. It also compares the molar ratios of angiogenic biomarkers in vascular (g) and lymphatic (h) invasions versus non‐invasive states in primary breast cancer patients. [Correction added on 15th July 2025, after first online publication: The previous sentence was added to the caption of Figure 1.] ALNM in *x*‐axis expresses ALNM ratio. Bars represent mean ± standard error. The ANG‐2 levels are presented in picograms per milliliter (pg/ml), while other biomarkers are measured in nanograms per milliliter (ng/ml). ^α^ *p* < 0.05.

The (ANG‐1 + ANG‐2)/VEGF‐C ratio was elevated in subjects with vascular invasion (VI) compared to those with non‐VI tumors (Figure 1, *p* < 0.01). While plasma ANG‐2 levels were also increased in tumors with LI^+^ compared to those without, this difference did not reach statistical significance (Figure 1). Furthermore, the mean ALNM ratio was higher in subjects with LI^+^ compared to those without (*p* < 0.01; Figure 1). Consistently, Table 2 illustrates the concordance between the ALNM ratio and LI, emphasizing the high accuracy of ALNM‐based classification.

**TABLE 2:** Associations between lymphatic‐ or vascular‐invasion and the involvement of axillary lymph node metastasis (ALMN‐negative or ‐positive).

	ALNM status		
	Positive	Negative	*p*
Lymphatic invasion			
Positive	41 (97.6%)	1 (2.4%)	
Negative	10 (35.7%)	18 (64.3%)	0.008
Vascular invasion			
Positive	49 (75.4%)	16 (24.6%)	
Negative	6 (40%)	9 (60%)	< 0.01

*Note:* The chi‐square test was performed. The data were expressed in the number of patients (percent).

### 3.3 Interactions Between Angiopoietins and VEGF‐C: Contributions to Lymphatic Anomalies

The multivariate models in Table 3 assessed how biomarkers contribute to VEGF variations, across pathologic status of ALNM, LI, and VI. Among participants with positive ALNM and LI, high plasma levels of lymphangiogenic biomarkers showed inverse correlations with VEGF‐C, including ANG‐2 (*β*__ALNM_ = − 0.35, *β*__LI_ = −0.34), ANG‐2/Tie‐2 (*β*__ALNM_ = −0.38, *β*__LI_ = −0.36), and ANGs/Tie‐2 (*β*__ALNM_ = −0.34, *β*__LI_ = −0.38) (*p* < 0.05). Additionally, an inverse correlation between ANGs/Tie‐2 and VEGF‐C was observed in participants without VI (*β* = −0.58, *p* < 0.05).

**TABLE 3:** Linear regression analysis to obtain correlation coefficients (*β*) between plasma levels of studied biomarkers and the corresponding ratio through stratification analyzes considering ALNM (+: involved and −: negative ALNM), vascular invasion, and lymphatic invasion status in unadjusted (crude), and adjusted models (*n* = 143.

	VEGF‐C (ng/mL)					
Variables^a^	ALNM+ (*n* = 88)	ALNM− (*n* = 55)	Lymphatic invasion (*n* = 63)	No lymphatic invasion (*n* = 41)	Vascular invasion (*n* = 90)	No vascular invasion (*n* = 26)
ANG‐1 (ng/mL)						
Crude	−0.15 (0.26)^b^	−0.02 (0.88)^b^	−0.17 (0.29)^b^	0.25 (0.20)^b^	−0.11 (0.39)^b^	0.27 (0.30)^b^
Adj. model^c^	−0.17 (0.28)	0.22 (0.31)	−0.21 (0.23)^e^	0.26 (0.22)^d^	−0.09 (0.52)	−0.40 (0.11)^d^
ANG‐2 (pg/mL)						
Crude	**−0.29 (0.03)** *	−0.02 (0.90)	−0.24 (0.14)	−0.20 (0.31)	−0.19 (0.16)	−0.30 (0.25)
Adj. model^c^	**−0.35 (0.03)** *	−0.05 (0.81)	**−0.34 (0.04)** *	−0.23 (0.25)	−0.25 (0.09)	−0.48 (0.08)
Tie‐2 (ng/mL)						
Crude	0.04 (0.74)	−0.20 (0.23)	0.05 (0.75)	−0.12 (0.53)	0.01 (0.99)	−0.07 (0.79)
Adj. model^c^	0.10 (0.51)	−0.07 (0.75)	0.13 (0.42)	−0.03 (0.85)	0.09 (0.51)	0.03 (0.90)
ANG‐1/ANG‐2						
Crude	0.15 (0.23)	0.19 (0.25)	0.28 (0.09)	0.13 (0.51)	0.17 (0.19)	−0.18 (0.50)
Adj. model^c^	0.22 (0.18)	0.28 (0.22)	0.26 (0.12)	0.18 (0.38)	0.21 (0.16)	−0.24 (0.27)
(ANG‐1 + ANG‐2)/Tie‐2						
Crude	**−0.28 (0.03)** *	0.01 (0.97)	−0.30 (0.06)	−0.02 (0.89)	−0.17 (0.20)	−0.33 (0.20)
Adj. model^c^	**−0.34 (0.03)** *	−0.01 (0.91)	**−0.38 (0.02)** *	−0.04 (0.83)	−0.25 (0.09)	**−0.58 (0.02)** *
ANG‐2/Tie‐2						
Crude	**−0.32 (0.01)** *	0.06 (0.73)	−0.29 (0.07)	−0.21 (0.28)	−0.14 (0.29)	−0.29 (0.26)
Adj. model^c^	**−0.38 (0.01)** *	−0.12 (0.60)	**−0.36 (0.03)** *	−0.24 (0.23)	**−0.30 (0.04)** *	−0.51 (0.06)

Abbreviations: ALNM, axillary lymph node metastasis; ANG‐1, angiopoietin‐1; ANG‐2, angiopoietin‐2; Tie‐2, tyrosine kinase with Ig and EGF homology domains 1; VEGF‐C, vascular endothelial growth factor‐C.

^a^ Some missing data exist in pathological variables.

^b^ Data were expressed as *β* (*p value*) in bold.

^c^ The model was adjusted for age at diagnosis (year) and lymphatic invasion (yes/no).

^d^ The model was adjusted for age at diagnosis (year) and body mass index (BMI) at diagnosis (kg/m^2^).

^e^ The model was adjusted for age at diagnosis (year) and axillary lymph node metastasis (yes/no).

* Statistical level of significance was considered in *p* < 0.05.

**FIGURE 2:**
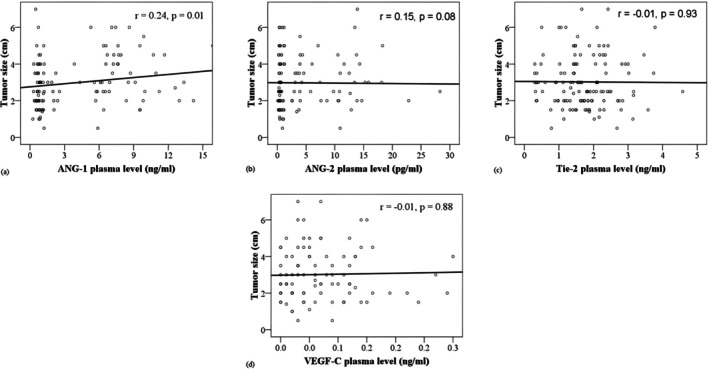
Correlations between angiogenic biomarkers and tumor size. Scatter plots illustrate the correlation between angiogenic plasma biomarkers and tumor size (cm) in primary breast cancer patients (*n* = 130). Pearson's correlation coefficient (*r*) is provided.

**FIGURE 3:**
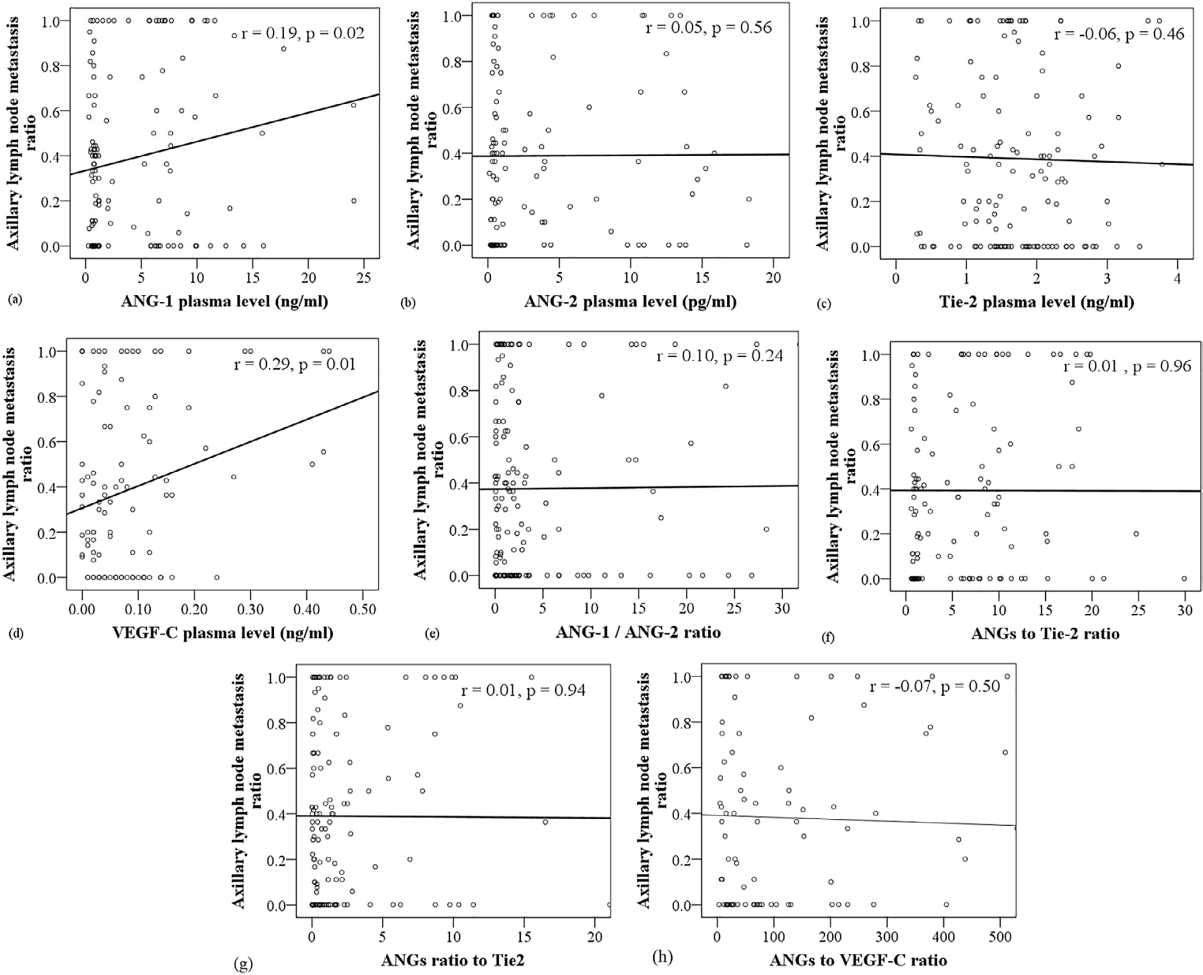
Correlations between plasma levels of angiogenic biomarkers and the ALNM ratio. Scatter plots depict individual angiogenic biomarkers (panels a–d) and ratios of angiogenic biomarkers (panels e–h) in relation to the axillary lymph node metastasis (ALNM) ratio, defined as the ratio of ALNM involvement to the total number of surgically dissected axillary lymph nodes, in primary breast cancer patients (*n* = 143). The ratio of ANGs to Tie‐2 represents the molar ratio (ANG‐1 + ANG‐2)/Tie‐2, while the ratio of ANGs to VEGF‐C represents the molar ratio (ANG‐1 + ANG‐2)/VEGF‐C.

Our findings revealed a significantly higher ALNM ratio in the LI^+^ group compared to tumors without LI (Figure 1), indicating consistency between these two variables. As illustrated in Figure 2, plasma ANG‐1 levels were correlated with tumor size (*r* = 0.24, *p* < 0.05). Figure 2 also illustrates a significant positive correlation between plasma ANG‐1 levels and tumor size (*r* = 0.19, *p* < 0.05). In Figure 3, correlations between pro‐angiogenic biomarkers and the ALNM ratio are depicted. Notably, plasma levels of ANG‐1 (*r* = 0.19) and VEGF‐C (*r* = 0.29) showed significant positive correlations with the ALNM ratio (*p* < 0.05).

### 3.4 Diagnostic Potential of Lymphangiogenic Biomarkers

**FIGURE 4:**
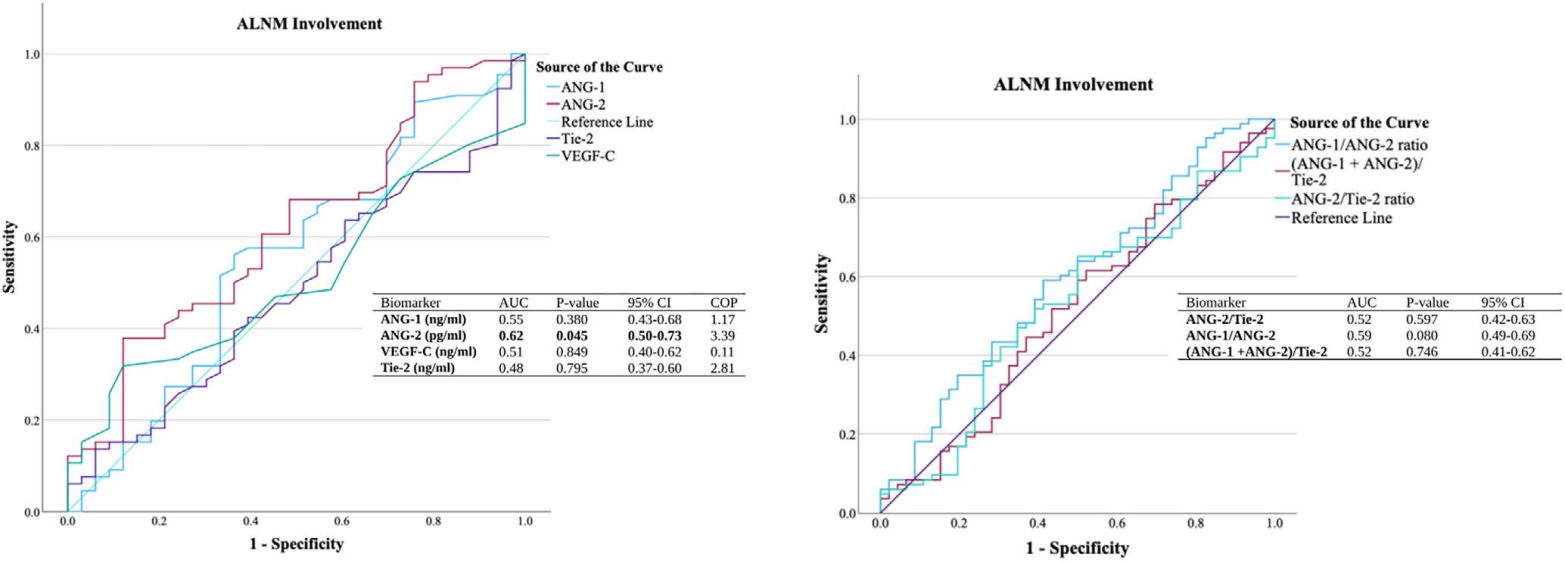
Diagnostic performance of lymphangiogenic biomarkers for ALNM. ROC curves and AUC analyzes of lymphangiogenic biomarkers associated with ALNM involvement (*n* = 143). The reference line represents the area of no diagnostic benefit (AUC = 50%) [40]. ANG‐1, angiopoietin‐1; ANG‐2, angiopoietin‐2; AUC, the are under the ROC curve; COP, cutoff point; ROC‐curve, receiver operating characteristic‐curve; Tie‐2, tyrosine kinase with Ig and EGF homology domains 1; VEGF‐C, vascular endothelial growth factor‐C.

**FIGURE 5:**
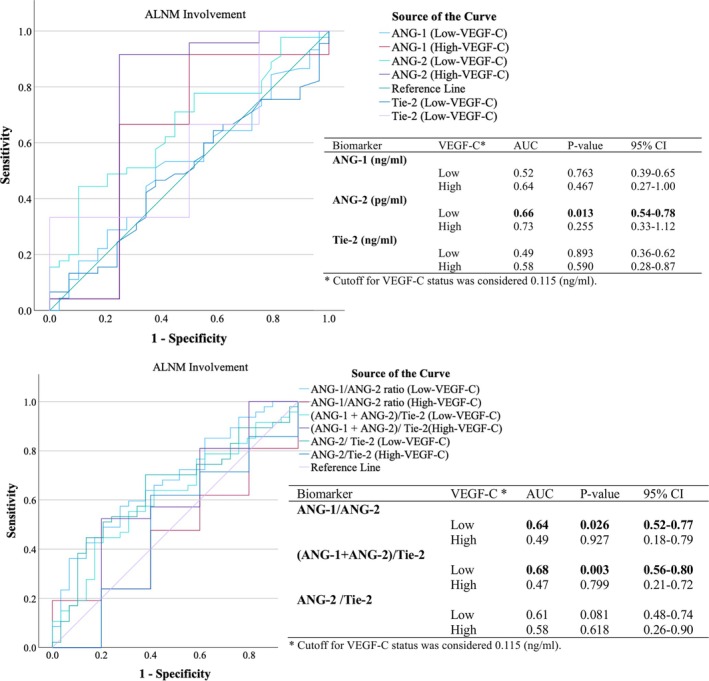
Diagnostic performance of lymphangiogenic biomarkers for ALNM in VEGF‐C subgroups. ROC curves and AUC analysis of lymphangiogenic biomarkers in correlation with ALNM involvement, stratified by VEGF‐C status. ANG‐1, angiopoietin‐1; ANG‐2, angiopoietin‐2; AUC, the are under the ROC curve; ROC‐curve, receiver operating characteristic‐curve; Tie‐2, tyrosine kinase with Ig and EGF homology domains 1; VEGF‐C, vascular endothelial growth factor‐C.

Figure 4 presents ROC curves that assess the diagnostic performance of angiogenic biomarkers for predicting ALNM status. The ANG‐2 demonstrated notable specificity and differentiation power for detecting ALNM positivity, achieving an AUC of 62% (*p* < 0.05). In comparison, the ANG‐1/ANG‐2 ratio showed a slightly lower AUC of 59% (*p* = 0.08). Figure 5 further highlighted that ANG‐2 performed particularly well in identifying ALNM involvement among patients with lower VEGF‐C levels (*p* < 0.05). Additionally, the ratios of (ANG‐1 + ANG‐2)/Tie‐2 and ANG‐1/ANG‐2 were found to be strong indicators of ALNM positivity, with significant AUCs ranging from 64% to 68%, especially in cases with low plasma VEGF‐C levels (*p* < 0.05). Linear regression analyzes (Table 4) revealed significant negative associations between the ANGs/Tie‐2 (*β* = −0.29) and ANG‐2/Tie‐2 (*β* = −0.28) ratios with VEGF‐C in Grade ІІ tumors (*p* < 0.05).

### 3.5 Receptor Status

Table S1 summarizes the hormonal receptor and HER‐2 statuses in relation to lymphatic and vascular invasions within the study population. Descriptively, a higher frequency of ER/PR expression was observed in patients with ALNM^+^ status, while HER‐2 negativity was more common in this group. Similarly, ER/PR expression was frequently observed in VI^+^ status. These findings were not statistically significant. Figure 1 shows that the average plasma levels of ANG‐2 were significantly higher in ER^+^ and PR^+^ patients compared to their HR‐negative counterparts (*p* < 0.05). However, no significant differences were observed in other angiogenic biomarkers based on tumoral HR status.

Table 5 summarizes the OR demonstrating associations between HR status and angiogenic biomarkers. Tumors exhibiting active PR signaling had a higher likelihood of elevated ANG‐2 levels (OR = 3.3, 95% CI: 1.01–10.8).

**TABLE 4:** Linear regression analysis to obtain correlation coefficients (*β*) between plasma levels of studied biomarkers and the corresponding ratios with the plasma level of VEGF‐C by considering the pathological grade of breast cancer patients in unadjusted (crude) and adjusted models^b^ (*N* = 139).

Variables	VEGF‐C (ng/ml)		
	Grade І (*n* = 16)	Grade ІІ (*n* = 112)	Grade ІІІ (*n* = 11)
ANG‐1 (ng/mL)			
Crude	0.67 (0.14)^a^	−0.13 (0.24)^a^	0.20 (0.66)^a^
Adj. model^b^	0.73 (0.20)	−0.17 (0.17)	−0.37 (0.65)
ANG‐2 (pg/mL)			
Crude	0.05 (0.91)	−0.21 (0.06)	0.20 (0.66)
Adj. model^b^	0.21 (0.70)	**−0.25 (0.04)** *	0.25 (0.80)
Tie‐2 (ng/mL)			
Crude	−0.41 (0.40)	0.06 (0.59)	−0.49 (0.25)
Adj. model^b^	−0.57 (0.32)	0.13 (0.29)	−0.09 (0.88)
ANG‐1/ANG‐2			
Crude	−0.50 (0.30)	0.21 (0.05)	−0.29 (0.51)
Adj. model^b^	−0.59 (0.31)	**0.24 (0.04)** *	0.47 (0.51)
ANG‐1 + ANG‐2/Tie‐2			
Crude	0.54 (0.26)	−0.20 (0.07)	0.32 (0.47)
Adj. model^b^	0.71 (0.13)	**−0.29 (0.01)** *	−0.01 (0.51)
ANG‐2/Tie‐2			
Crude	0.01 (0.99)	−0.15 (0.17)	0.11 (0.80)
Adj. model	0.15 (0.79)	**−0.28 (0.02)** *	0.34 (0.73)

*Note:* Some missing data exist in pathological grades. * Statistical level of significance was considered in *p* < 0.05.

Abbreviations: ANG‐1, angiopoietin‐1; ANG‐2, angiopoietin‐2; *n*, number of participants; Tie‐2, tyrosine kinase with Ig and EGF homology domains 1; VEGF‐C, vascular endothelial growth factor‐C.

^a^ Data were expressed as *β* (*p* value) in bold.

^b^ The model was adjusted for age at diagnosis (year) and body mass index (BMI) at diagnosis (kg/m^2^).

**TABLE 5:** Odds ratios (OR) and 95% confidence intervals (CI) of hormonal receptor status (as independent variables) in association with the levels of angiopoietins, Tie‐2, and VEGF‐C (as dependent data) in the study population (*N* = 149).

	ER			PR			HER2		
Variables^a^	Positive (*n* = 136)	Negative (*n* = 13)	OR (95% CI)^b^	Positive (*n* = 131)	Negative (*n* = 17)	OR (95%CI)	Positive (*n* = 29)	Negative (*n* = 120)	OR (95%CI)
ANG‐1 (ng/mL)									
Mean ± S.D.	4.44 ± 5.29	3.32 ± 4.14		4.42 ± 5.26	3.92 ± 4.87		5.82 ± 7.65	3.95 ± 4.31	
< 1.19	65 (90.3)	7 (9.7)	1.00	62 (87.3)	9 (12.7)	1.00	15 (20.8)	57 (79.2)	1.00
≥ 1.19	64 (91.4)	6 (8.6)	1.1 (0.3–3.6)	62 (88.6)	8 (11.4)	1. 1 (0.4–3.1)	14 (20.0)	56 (80.0)	0.9 (0.4–2.1)
ANG‐2 (pg/mL)									
Mean ± S.D.	4.21 ± 5.66	2.31 ± 3.57		4.36 ± 5.74	1.92 ± 3.14		2.77 ± 4.27	4.36 ± 5.77	
< 0.88	61 (87.1)	9 (12.9)	1.00	58 (82.9)	12 (17.1)	1.00	18 (25.7)	52 (74.3)	1.00
≥ 0.88	66 (95.7)	3 (4.3)	3.2 (0.8–12.5)	64 (94.1)	4 (5.9)	**3.3 (1.01–10.8)** *	10 (14.5)	59 (85.5)	0.4 (0.1–1.0)
Tie‐2 (ng/mL)									
Mean ± S.D.	1.73 ± 0.85	1.47 ± 0.79		1.71 ± 0.85	1.66 ± 0.90		1.74 ± 0.98	1.70 ± 0.81	
< 1.64	65 (89.0)	8 (11.0)	1.00	63 (87.5)	9 (12.5)	1.00	15 (20.5)	58 (79.5)	1.00
≥ 1.64	65 (92.9)	5 (7.1)	1.6 (0.4–5.1)	62 (88.6)	8 (11.4)	1.1 (0.4–3.0)	14 (20.0)	56 (80.0)	0.9 (0.4–2.1)
VEGF‐C (ng/mL)									
Mean ± S.D.	0.08 ± 0.09	0.10 ± 0.08		0.08 ± 0.08	0.11 ± 0.11		0.06 ± 0.05	0.08 ± 0.09	
< 0.06	55 (91.7)	5 (8.3)	1.00	53 (89.8)	6 (10.2)	1.00	14 (23.3)	46 (76.7)	1.00
≥ 0.06	49 (89.1)	6 (10.9)	0.7 (0.2–2.5)	47 (85.5)	8 (14.5)	0.6 (0.2–2.0)	9 (16.4)	46 (83.6)	0.6 (0.2–1.6)

*Note:* The classification of ANG‐1, ANG‐2, Tie‐2, and VEGF‐C were based on the median plasma levels of our studied population.

Abbreviations: ANG‐1, angiopoietin‐1; ANG‐2, angiopoietin‐2; ER, estrogen receptor, HER2, human epidermal growth factor receptor 2, PR; progesterone receptor, Tie‐2, tyrosine kinase with Ig and EGF homology domains 1; VEGF‐C; vascular endothelial growth factor‐C.

^a^ All variables were categorized into dichotomous groups.

^b^ OR (95%CI) was obtained by the chi‐square test.

* Statistical level of significance was considered in *p* < 0.05.

Table 6 displays correlations between independent biomarkers (ANG‐1, ANG‐2, and Tie‐2) and plasma VEGF‐C levels based on dichotomous HR status in breast tumors. Plasma ANG‐1 levels exhibited a significant inverse correlation with VEGF‐C among HER‐2^+^ patients in both crude (*β* = −0.46) and adjusted (*β* = −0.61) models (*p* < 0.05). The inverse correlations between ANGs/Tie‐2 ratio and VEGF‐C in the HER‐2^+^ subgroup were observed in both the crude (*β* = −0.52, *p* < 0.05) and adjusted models (*β* = −0.57, *p* < 0.05). A significant positive correlation was attained between the ANG‐1/ANG‐2 ratio and plasma VEGF‐C among ER^+^ patients in the adjusted model (β = 0.22, *p* < 0.05). An inverse relationship between ANG‐2/Tie‐2 and VEGF‐C in the ER^−^ subgroup of breast cancer patients was also evident (β = −0.61, *p* < 0.05).

**TABLE 6:** Linear regression analysis to obtain correlation coefficient (*β*) between the plasma level of studied biomarkers and the plasma level of VEGF‐C by considering hormonal receptor status in non‐adjusted (crude) and adjusted^b^ models (*N* = 149).

	VEGF‐C (ng/ml)					
Variables	ER+ (*n* = 136)	ER− (*n* = 13)	PR+ (*n* = 131)	PR− (*n* = 17)	HER2+ (*n* = 29)	HER2− (*n* = 120)
ANG‐1 (ng/mL)						
Crude	−0.02 (0.79)^a^	−0.34 (0.30)^a^	−0.07 (0.47)^a^	0.04 (0.86)^a^	**−0.46 (0.04)** ^a^, *	0.04 (0.69)^a^
Adj. model^b^	−0.05 (0.63)^a^	−0.37 (0.36)	−0.10 (0.33)	0.03 (0.91)	**−0.61 (0.01)** a, *	0.02 (0.84)
ANG‐2 (pg/mL)						
Crude	−0.12 (0.24)	**−0.79 (0.01)** a, *	−0.13 (0.20)	−0.39 (0.16)	−0.35 (0.12)	−0.12 (0.25)
Adj. model^b^	−0.12 (0.28)	**−0.88 (0.01)** a, *	−0.13 (0.23)	−0.53 (0.11)	−0.35 (0.19)	−0.13 (0.26)
Tie‐2 (ng/mL)						
Crude	0.05 (0.57)	−0.46 (0.15)	0.06 (0.53)	−0.28 (0.32)	−0.16 (0.50)	0.06 (0.56)
Adj. model^b^	0.09 (0.38)	−0.48 (0.25)	0.10 (0.36)	−0.27 (0.44)	−0.23 (0.55)	0.10 (0.38)
ANG‐1/ANG‐2						
Crude	0.19 (0.05)	0.06 (0.84)	0.11 (0.28)	0.43 (0.11)	0.35 (0.12)	0.16 (0.14)
Adj. model^b^	**0.22 (0.04)** a, *	0.08 (0.87)	0.14 (0.19)	0.43 (0.18)	0.46 (0.08)	0.17 (0.12)
ANG‐1 + ANG‐2/Tie‐2						
Crude	−0.11 (0.27)	−0.52 (0.10)	−0.13 (0.19)	−0.24 (0.40)	**−0.52 (0.01)** a, *	−0.08 (0.42)
Adj. model^b^	−0.14 (0.18)	−0.58 (0.14)	−0.17 (0.11)	−0.28 (0.39)	**−0.57 (0.02)** a, *	−0.11 (0.30)
ANG‐2/Tie‐2						
Crude	−0.12 (0.25)	**−0.61 (0.04)** a, *	−0.12 (0.23)	−0.43 (0.11)	−0.43 (0.05)	−0.12 (0.27)
Adj. model^b^	−0.15 (0.17)	−0.77 (0.05)	−0.16 (0.15)	−0.52 (0.11)	−0.41 (0.12)	−0.15 (0.17)

Abbreviations: ANG‐1, angiopoietin‐1; ANG‐2, angiopoietin‐2; ER, estrogen receptor; PR, progesterone receptor; HER2, human epidermal growth factor receptor 2; Tie‐2, tyrosine kinase with Ig and EGF homology domains 1; VEGF‐C, vascular endothelial growth factor‐C.

^a^ Data were expressed as *β* (*p value*) in bold.

^b^ The model was adjusted for age at diagnosis (year) and body mass index (BMI) at diagnosis (kg/m^2^).

* Statistical level of significance was considered in *p* < 0.05.

## 4 Discussion

Our findings revealed a significant inverse correlation between the ANGs‐Tie‐2 ratio and VEGF‐C levels in patients with grade II breast tumors. Based on ROC analysis, this study is the first to demonstrate that ANG‐2 is a remarkable biomarker for assessing ALNM status, particularly in patients with low VEGF‐C levels. Notably, the accuracy of this assessment improved further when the ANGs/Tie‐2 ratio was incorporated in patients with low VEGF‐C. Elevated plasma ANG‐2 levels were found in ER^+^ tumors, and higher ANGs/VEGF‐C ratios were noted in VI+ cases. Additionally, HER‐2^+^ tumors showed an inverse correlation between ANG‐1 and VEGF‐C, suggesting receptor‐dependent variations in angiogenic markers.

### 4.1 Lymphangiogenic Biomarkers

There was a strong correlation between plasma VEGF‐C levels, and the ALNM ratio, aligning with previous research that could further highlight the critical role of VEGF‐C in promoting lymphangiogenic metastasis in invasive breast cancer [6, 42]. VEGF‐C stands out as a key factor in lymphovascular formation [6, 8]. However, its interaction with ANGs is crucial for influencing lymphovascular sprouting and vessel diameter [13, 43].

In addition to the well‐established antagonistic roles of ANG‐1 and ANG‐2 in vascular formation, recent studies have highlighted synergistic contribution of other pro‐angiogenic factors, beside ANG function, to lymphangiogenesis [20, 44]. The AUC analysis demonstrated that ANG‐2 exhibited a significant ability to more accurately assess ALNM status, underscoring its potential as a reliable biomarker for metastatic progression. The ROC curve identified an optimal cutoff for ANG‐2 at 3.39 pg/mL for detecting ALNM status. This cutoff is above the reference range reported in a previous study involving non‐cancerous conditions, such as hypertension [41]. Furthermore, ANG‐2 effectively identified ALNM status in 66% of breast cancer patients with low VEGF‐C levels. The interaction of VEGF‐C with ANG‐2 overregulation is essential for endothelial and pericyte cell growth, which seems crucial for lymphangiogenesis [45]. The VEGF‐C has been shown to activate lymphatic endothelial cells, leading to increased ANG‐2 transcription [46]. Consequently, ANG‐2 plays a central role in both the maturation of new vascular formation and lymphatic angiogenesis [47]. In vivo mouse studies supported the role of ANG‐2 in lymphangiogenesis in malignancies of the small intestine and skin [46]. Imanishi et al. showed that ANG‐2 activates α5β1 integrin/integrin‐linked kinase‐Akt‐GSK‐3β‐Snail pathway, promoting metastasis to distant organs [48]. The present findings from the AUC analysis demonstrated a 68% improvement in detection rate when incorporating the ANGs/Tie‐2 ratio, particularly in patients stratified based on low VEGF‐C levels. This combination of biomarkers improved the accuracy of identifying ALNM positivity in these patients compared to using individual markers alone.In ANG‐2‐deficient mice, impaired lymphatic vascular function and formation were observed, which was restored by ANG‐1, suggesting a coordinating role for ANG‐1 and ANG‐2 in regulating lymphangiogenesis and maintaining lymphatic homeostasis [44, 49]. In the present study, the plasma level of ANG‐1 was positively correlated with the ALNM ratio, highlighting its significance in lymphatic anomalies and increasing the ALNM ratio. Previous in vitro and in vivo studies have elucidated the essential contribution of ANG‐1 to the proliferation of lymphatic endothelial cells, as observed in mouse cornea studies [49]. The intricate role of ANG in conjunction with other angiogenic biomarkers in breast cancer‐associated lymphatic anomalies, along with the involvement of additional transcriptional factors in regulating lymphangiogenesis, underscores the importance of identifying key mediators in the ANGs‐VEGFs signaling pathway.

This study highlighted the importance of angiogenic factors in invasive subtypes of breast cancer (ALNM, VI^+^, and LI^+^), particularly the interplay between ANGs and VEGF‐C. Our findings demonstrated a higher ANGs/VEGF‐C ratio in patients with VI^+^. Consistent with this, in vitro and animal studies have shown that ANG‐2 overexpression, dependent on VEGF expression status, is linked to increased endothelial cell proliferation, vascular infiltration, and a greater likelihood of metastasis [48]. Additionally, our findings of inverse correlations between ANG‐2 and VEGF‐C in patients with ALNM and LI^+^ tumors could suggest that a combination of pro‐angiogenic factors, rather than VEGF‐C alone, might more effectively differentiate lymphovascular anomalies [27]. While this study sheds light on the interplay of angiogenic factors in pathological contexts of lymphatic anomalies, it highlights the necessity for biomarker validation through large‐scale prospective epidemiological studies.

### 4.2 Pathological and Hormonal Receptor Status

The present findings demonstrated plasma ANG‐2 levels were lower in ER^−^ and PR^−^ statuses, suggesting a potential role for HR‐induced regulations of ANG‐2 expression. In ER^−^ patients, notable inverse correlations were observed between VEGF‐C and both ANG‐2 and the ANG‐2/Tie‐2 ratio, suggesting that VEGF‐C might inversely influence ANG‐2 levels. Conversely, in ER^+^ patients, significant positive relationships were found between VEGF‐C and ANG‐1/ANG‐2, indicating that VEGF‐C could contribute to ANG‐1 transcription, potentially influenced by the upstream activity of ER across other molecular pathways. Additionally, Harfouche et al. demonstrated that ER upregulation led to *ANGPT‐2* overexpression, which was negatively associated with *ANGPT1* mRNA expression in breast cancer cell lines [24]. Despite HR‐dependent changes in ANG levels, VEGF‐C levels did not differ between HR statuses. This suggests that HR status might act as a key modifier, potentially altering the relationship between VEGF‐C and ANG‐2 to an inverse correlation. Although some studies have suggested the presence of a functional estrogen‐sensitive response element in the regulatory region of the *VEGF* genes [50], our findings could not support ER‐dependent variations in VEGF‐C levels. Our observations of ER‐dependent correlations between VEGF‐C and ANG‐1/ANG‐2 could highlight the potential for further experimental research to explore how estrogen and its antagonists might directly influence the production of key angiogenic factors in hormone‐responsive tumors. Overall, our findings suggest that VEGF‐C‐dependent variations in ANG levels are predominantly associated with tumoral ER status [26]. These findings warrant future studies with clinical implications, particularly exploring the administration of ER antagonists to suppress lymphangiogenesis in breast cancer.

Active and overexpressed HER‐2 in tumors is closely associated with poorer survival outcomes in breast cancer patients [51]. Our results indicated a potential inverse correlation between ANGs/Tie‐2 ratio and VEGF‐C levels, suggesting transcriptional upregulation of *ANGPT* genes, particularly among HER‐2‐positive breast cancer patients. In addition, *ANG‐1* levels showed a significant inverse correlation with VEGF‐C levels, particularly in patients with HER‐2 positive tumors. Niu and Carter highlighted the HER‐2 signaling in activating AKT and extracellular regulated kinase (ERK)/mitogen‐activated protein kinase (MAPK), leading to ANGPT upregulation [52]. Additionally, Schoppmann et al. demonstrated that HER‐2 stimulation induces *VEGF‐C* upregulation in the MCF‐7 cell line, thereby increasing the risk of lymphatic metastasis in tumoral breast cancer cells [9]. While correlations between ANGs and VEGF‐C based on HER‐2 staus have been noted, understanding the clinical importance of this triple‐axis pathway is crucial for guiding future research into lymphatic diagnosis and treatments to prevent lymphoangiogenesis in breast cancer.

In grade II disease, VEGF‐C levels exhibited inverse correlations with plasma levels of ANG‐2/Tie‐2, and ANGs/Tie‐2, suggesting that ANGs, and their specific receptor, Tie‐2, are involved in events closely related to VEGF‐C regulation. Conversely, the correlation was positive for the ANG‐1/ANG‐2 ratio, indicating a direct relationship with ANG‐1, whereas for ANG‐2, VEGF‐C can function as an inverse modulator, contributing to the upstream regulatory effects of VEGF‐C. Several epidemiologic studies have reported the overexpression of *ANGPT2* across various stages of carcinogenesis [53, 54]. In this context, Caine et al. demonstrated elevated plasma levels of VEGF and Tie‐2 in both breast and prostate cancers compared to controls [53], highlighting a meaningful interrelationship between Tie‐2‐related angiogenic growth [53]. These findings collectively suggest that angiogenic factors might influence cancer progression by affecting the invasive phenotype of malignant cells [48]. In addition to the integration of VEGF‐C to contain the dynamic balance between ANG‐1 and ANG‐2 (ANG‐1/ANG‐2) [13, 14], the findings suggested that high VEGF‐C levels, correlating with ANG‐2 dysregulation, may enhance lymphangiogenesis and promote vascular invasiveness phenotypes [11]. [Correction added on 15th July 2025, after first online publication: In the previous sentence, the text “correlating with ANG‐2 upregulation,” was corrected to “correlating with ANG‐2 dysregulation,”.] However, the observed positive correlations between ANG‐1/ANG‐1 and VEGF‐C in vascular invasive subgroup analyzes require a larger sample size to achieve statistical significance.

Overall, the ANGs/Tie‐2/VEGF‐C axis emerged as a prominent biomarker panel across breast cancer molecular subtypes, including ER/PR or HER‐2 statuses. These key pro‐angiogenic biomarkers provide valuable insights for future research focused on developing diagnostic platforms to assess the effects of antagonistic components targeting estradiol and EGF. Such studies could also inform strategies aimed at preventing lymphangiogenesis in breast cancer.

The study had some limitations that should be acknowledged. The sample size was a constraint, particularly for analyzing specific subgroups. Additionally, most of our study population was diagnosed with grade II, which may influence the interpretation of the significant results obtained. Therefore, caution is advised when interpreting these findings.

## 5 Conclusions

Our findings reveal inverse correlations between VEGF‐C and angiopoietins (ANG‐2, ANG‐2/Tie‐2, and ANG‐1 + ANG‐2/Tie‐2) in breast cancer patients with tumor grade II. Notably, HER‐2^+^‐dependent inverse relationships were observed between VEGF‐C and ANGs, including ANG‐1 and the (ANG‐1 + ANG‐2)/Tie‐2 ratio. Additionally, the results suggested that ER‐PR expression status might influence ANG‐2 upregulation. The study highlighted the significant associations between the ANG/Tie‐2 axis, and VEGF‐C, contributing to lymphatic anomalies (i.e., ALNM and lymphatic invasiveness). The ROC curve analysis demonstrated the significant performance of the ANG‐1/ANG‐2 and (ANG‐1 + ANG‐2)/Tie‐2 ratios in detecting ALNM involvement, particularly in patients with low VEGF‐C levels. A similar result was observed with ANG‐2. Understanding the complex interaction among pro‐angiogenic factors (ANG/Tie‐2/VEGF‐C) and their relationship with ER or HER‐2 status underscores the clinical relevance of these biomarkers in detecting ALNM in breast cancer patients.

## Author Contributions

Conceptualization, S.P.; data curation, V.M., A.F., and S.P.; formal analysis, S.P., P.V., and A.F.; funding acquisition, and project administration, S.P.; resources, V.M., A.F., and S.P.; investigation, V.M., P.V., and S.P.; methodology, S.P. and V.M., supervision and validation, S.P. and V.M.; visualization, writing – original draft, S.P., V.M., and P.V.; writing – review and editing, S.P. and V.M.

## Ethics Statement

The procedures and protocols of the study were approved by the Ethical Committee of Tabriz University of Medical Sciences, Tabriz, Iran (Ethical code: 5‐4‐8327).

## Conflicts of Interest

The authors declare no conflicts of interest.

## Supporting information


**Table S1.** Descriptive analysis presenting hormonal receptors and HER‐2 status in differentiating vascular and lymphatic anomalies among breast cancer patients.

## Data Availability

The data that support the findings of this study are available from the corresponding author upon reasonable request.
