# Correction to “Hormone Receptor‐Dependent Correlations Between Angiopoietins and VEGF‐C in Primary Breast Cancer: Insights Into Lymphangiogenic Biomarkers”

**DOI:** 10.1002/cnr2.70252

**Published:** 2025-07-02

V. Montazeri , P. Varshosaz , A. Fakhrjou , and S. Pirouzpanah , “Hormone Receptor‐Dependent Correlations Between Angiopoietins and VEGF‐C in Primary Breast Cancer: Insights Into Lymphangiogenic Biomarkers,” Cancer Reports 8, no. 5 (2025): e70101, 10.1002/cnr2.70101.40348609 PMC12063722

The following changes have been made to the above‐mentioned article:

– The affiliations of authors Vahid Montazeri and Ashraf Fakhrjou were corrected.
– The caption of Figure 1 was corrected to include explanations of panels g and h.
– In paragraph 3 of the section “4.2 Pathological and Hormonal Receptor Status”, the text “correlating with ANG‐2 upregulation,” was corrected to “correlating with ANG‐2 dysregulation,”.

These changes can be seen in the published version.

The publisher apologizes for these errors.

