# Supplementary material for: Hormone Receptor‐Dependent Correlations Between Angiopoietins and VEGF‐C in Primary Breast Cancer: Insights Into Lymphangiogenic Biomarkers
Source: Cancer Rep (Hoboken). 2025 May 9;8(5):e70101. doi: 10.1002/cnr2.70101 (PMC12063722; doi:10.1002/cnr2.70101)
Supplement: Supplementary file 1 — Table S1. Descriptive analysis presenting hormonal receptors and HER‐2 status in differentiating vascular and lymphatic anomalies among breast cancer patients. [file CNR2-8-e70101-s001.docx]

**Supplementary Table 1.** Descriptive analysis presenting hormonal receptors and HER-2 status in differentiating vascular and lymphatic anomalies among breast cancer patients.

|  | **ALNM** | | **LI** | | **VI** | |
| --- | --- | --- | --- | --- | --- | --- |
| Receptor status | - | + | - | + | - | + |
| **ER** |  |  |  |  |  |  |
| - | 3 (6.4) | 10(10.9) | 1(3.6) | 5(11.9) | 0(0.0) | 8(12.3) |
| + | 44(93.6) | 82(89.1) | 27(96.4) | 37(88.1) | 15(100.0) | 57(87.7) |
| *P-value* |  | 0.543* |  | 0.390* |  | 0.341* |
|  |  |  |  |  |  |  |
| **PR** |  |  |  |  |  |  |
| - | 5(10.6) | 12(13.2) | 3(10.7) | 6(14.6) | 2(13.3) | 8(12.5) |
| + | 42(89.4) | 79(86.8) | 25(89.3) | 35(85.4) | 13(86.7) | 56(87.5) |
| *P-value* |  | 0.789* |  | 0.729* |  | 1.000 |
|  |  |  |  |  |  |  |
| **Her-2** |  |  |  |  |  |  |
| - | 40(85.1) | 71(77.2) | 23(82.1) | 33(78.6) | 12(80.0) | 52(80.0) |
| + | 7(14.9) | 21(22.8) | 5(17.9) | 9(21.4) | 3(20.0) | 13(20.0) |
| *P-value* |  | 0.270 |  | 0.770* |  | 0.623* |

ALNM, axillary lymph node metastases; LI, lymphovascular invasions; VI, vascular invasion; ER, estrogen receptor; PR, progesterone receptor; HER-2, human epidermal growth factor receptor 2.

* The *P-value* was obtained by the Fisher’s exact test.
